# Adsorption Characteristics of Ionic Surfactants on Anthracite Surface: A Combined Experimental and Modeling Study

**DOI:** 10.3390/molecules27165314

**Published:** 2022-08-20

**Authors:** Xuyang Bai, Guochao Yan, Xuanlai Chen, Jiajun Li

**Affiliations:** 1 School of Mining Engineering, Taiyuan University of Technology, Taiyuan 030024, China; 2 School of Engineering, The University of Western Australia, Perth, WA 6009, Australia

**Keywords:** ionic surfactant, anthracite, adsorption characteristics

## Abstract

Ionic surfactants are widely used in coal dust control in mines, and their adsorption characteristics on the coal surface have a great influence on the coal dust control effect. In this investigation, anionic sodium dodecylbenzenesulfonate (SDBS) and cationic octadecyltrimethylammonium chloride (STAC) were selected to explore the adsorption characteristics of ionic surfactants on the surface of anthracite. The experimental results show that the adsorption rate and efficiency of STAC on the surface of anthracite are higher than that of SDBS; STAC can form a denser surfactant layer on the surface of anthracite, with a larger adsorption capacity and higher strength. Molecular dynamics simulations show that the adsorption between STAC and the surface of anthracite is tighter, and the distribution at the coal–water interface is more uniform; the surface of anthracite modified by STAC has a stronger binding ability to water molecules.

## 1. Introduction

As one of the most important primary energy sources in the world, coal occupies an irreplaceable position [1,2]. In the process of mining, storing and transporting coal resources, a large amount of coal dust will be generated, which causes serious damage to the environment [3,4,5]; at the same time, some coal mines contain inhalable and respirable coal dust content at rates of as high as 300 mg/m^3^ [6], which will endanger the life and health of workers [7,8]. In addition, when the concentration of coal dust reaches a certain level, dust explosions may occur, which seriously hinder the development of the social economy [9]. The management of coal dust is important. At present, most coal mines are still treated by simple water spray. However, water has high surface tension, and it is difficult for inhalable and respirable coal dust to be wetted in a short time [10,11,12,13]. High-rank coal dust, as represented by anthracite, is extremely difficult to wet [14,15]. The reason for this is that the anthracite has a high degree of coalification, a large carbon content, and a very low oxygen content. The overall performance is hydrophobic, and it is difficult to wet by water to achieve settlement [16,17,18]. In recent years, many researchers have found that adding chemicals such as surfactants to water can significantly reduce the surface tension of water and physically modify the hydrophobic surface of coal dust, thereby enhancing the wettability of coal dust [13,19,20].

Zhang et al. [21] found that the hydrophilicity of coal first increased and then decreased with the increase in carbon content through XPS, contact angle and molecular dynamics simulation studies. You et al. [22] studied the dehydration process of surfactant TX-100 (TX-100 was obtained from DOW Chemical Company, and is a branched-chain oc-tylphenol with 9.5 moles of ethylene oxide per molecule and a critical micelle concentration (CMC) of 189 mg/L at 25 °C) in lignite through dehydration experiments and MD simulation methods. The results showed that TX-100 would undergo monomolecular adsorption on the surface of lignite, making lignite more hydrophobic. XIA et al. [23] studied the adsorption of cationic surfactants on the coal surface from a microscopic perspective by combining experimental tests and molecular dynamics simulations. It was found that the polar head group shields the hydrophilic sites on the coal surface and enhances the hydrophobicity of the coal. The research by GUI and XIA et al. [24,25] found that the polar functional groups in the surfactants had a certain water-molecule bridging effect on the hydrogen bonds of the hydrophilic sites on the coal surface, so that the surfactants were tightly adsorbed on the coal surface. XIA et al. [26] studied the adsorption characteristics of DTAB on the coal surface through experiments and molecular dynamics simulation methods. The study showed that high concentrations of dodecyltrimethylammonium bromide would form a bilayer or micelle adsorption on the coal surface. Through XPS and molecular dynamics simulations, He et al. [27] showed that the adsorption process of nonylphenol ethoxylate on the lignite surface was spontaneous, and the polar interaction between the two was the main factor affecting the adsorption behavior. Chen et al. [28] constructed a 55-component macromolecular coal model, and studied the effect of the branched-chain structure in the polyoxyethylene unit on the wettability of coal by combining MD simulation and experiments. The results showed that the branched structure in the polyoxyethylene unit could make the coal surface more hydrophilic.

The adsorption characteristics of surfactants on the surface of coal substantially influence wettability. However, the tiny adsorption of ionic surfactants on the anthracite surface is seldom investigated. In this study, experiments and molecular dynamics techniques were used to examine the adsorption characteristics of the surfactants STAC and SDBS on the surface of anthracite.

## 2. Experiments and Simulations

### 2.1. Materials

**Figure 1:**
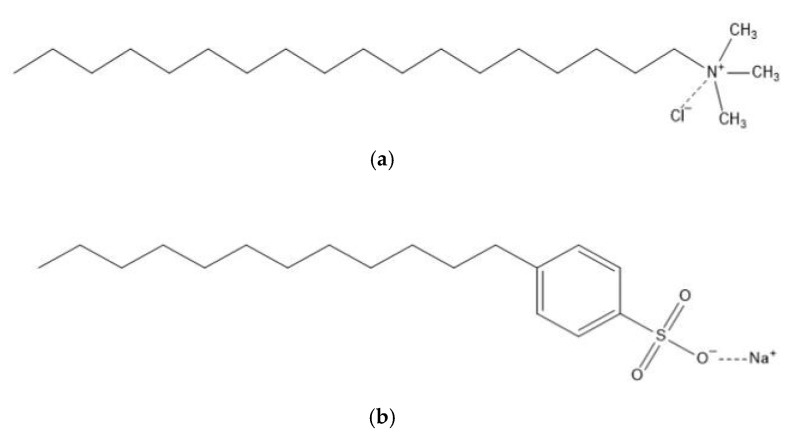
(**a**) STAC molecular structure; (**b**) SDBS molecular structure.

In this study, the underground No. 3 coal seam (Jincheng anthracite) was selected from the Zhaozhuang Coal Mine in Jincheng, Shanxi, China. The coal blocks were put into a crusher and crushed, and the 8411 electric vibrating screen was used to screen the coal into 200–300 mesh samples. At room temperature, HCl/HF/HCl acid ash removal treatment was performed, and the ash content after acid washing was 0.3%. The treated coal samples were dried in a vacuum-drying oven at 398 K for 2 h [29,30]. The cationic surfactant, octadecyltrimethyl chloride (STAC) (analytical grade, purity ≥ 99%, CAS: 112-03-8) was used in this study, and its molecular structure is shown in Figure 1a. Anionic surfactant is sodium dodecylbenzenesulfonate (SDBS) (analytical grade, purity ≥ 99%, CAS: 25155-30-0); its molecular structure is shown in Figure 1b. The above two agents were purchased from Shanghai Aladdin Biochemical Technology Co., Ltd. in Shanghai, China.

The concentration of SDBD and STAC solution is calculated and configured according to Formula (1).

(1) m=M × c × ω × 0.1

where M is the molar mass; c is the molar concentration; ω is the surfactant mass fraction.

### 2.2. Experiment

#### 2.2.1. Surfactant Adsorption

At room temperature, the critical micelle concentrations (CMC) of SDBS are about 2.30 mmol/L, and the STAC is about 3.20 mmol/L [31,32,33]. To ensure the sufficient adsorption of surfactants on the surface of anthracite [34,35,36,37], the concentrations of the two surfactants selected in this experiment were both 2000 mg/L, the molar concentration of SDBS was 5.7394 mmol/L, and the molar concentration of STAC was 5.7463 mmol/L, which were higher than their respective CMC.

A total of 400 mg of anthracite samples and 400 mL of surfactant solution were placed in a 500 mL beaker, and the beaker was placed in a constant-temperature magnetic stirrer for 10 h under specific conditions of 298 K and 800 r/min. The obtained solution was centrifuged at 2000 r/min for 20 min in a centrifuge; then, the supernatant was discarded and the samples were washed with deionized water, vacuum-filtered 3 times, and dried under vacuum at 378 K for 2 h.

#### 2.2.2. Contact Angle Measurement

A 200 mg coal sample was weighed with an electronic balance, and the pulverized coal was kept under a pressure of 20 MPa for 5 min by a tablet machine, and then taken out to make a circular coal piece with a diameter of 12 mm and a thickness of about 2 mm. JY-82C contact angle measuring instrument was used to measure the contact angles of coal samples with distilled water and two surfactant monomer solutions. The contact angle was measured using the pulverized coal method, and the principle of parallel experiments was adopted. Each experiment was conducted three times, and the average value of the three groups of data was taken.

#### 2.2.3. XPS Measurements

The experiments were performed using a Thermo Scientific K-Alpha X-ray electron spectrometer Which was purchased from Thermo Fisher Scientific Corporation in Shanghai, China. A monochromatic Al target (Kα hν = 1486.6 eV) was used as the X-ray source. During the test, the vacuum degree of the analysis chamber was better than 5.0 × 10^−7^ mBar. The full-spectrum scan pass energy was 100 eV, and the resolution of the measurement scan was 1 eV. The narrow-spectrum scan was performed for 5 cycles of signal accumulation (different scan times for different elements); the pass energy was 50 eV, and the step size was 0.05 eV, and these were used to scan C 1s, O 1s and N 1s. The binding energy was charge-corrected for the spectrum by contaminating carbon (C 1s = 284.8 eV) [38]. Background effects were subtracted using Shirley’s method. The experimental results were analyzed using Avantage software for peak fitting and data processing [29,39].

#### 2.2.4. FTIR Measurements

A Thermo Scientific Nicolet iS20 Fourier transform infrared spectrometer was used for the experiments. It was purchased from Thermo Fisher Scientific Corporation in Shanghai, China. Before the experiment, the raw coal samples, the samples after SDBS and STAC adsorption with anthracite, and KBr solid powder reagents were dried in a vacuum-drying oven to remove moisture, and the influence of moisture on the infrared test was excluded [40,41]. A 2 mg powder sample and 200 mg pure KBr were ground according to the ratio of 1:100. The sample was pressed into a transparent sheet on a powder tableting machine at a pressure of 10 Mpa, and the sample was placed in a Fourier transform infrared spectrometer for testing. The wavenumber range was 4000–400 cm^−1^, the number of scans was 32, the resolution was 4 cm^−1^, and the moving mirror speed was 0.4747. To reduce errors, the experimental results were automatically corrected and data-processed using OMNIC software [41,42,43].

#### 2.2.5. Molecular Dynamics Simulation

In this investigation, the anthracite model proposed earlier by our team was selected (C_7730_H_3916_O_133_N_123_S_25_) [44]. The unit cell size of the anthracite model was 5.4 × 4.8 × 5.5 nm^3^ (x·y·z), and the model density was 1.43 g/cm^3^. For the parameter settings of the simulation, we refer to our previous work [28]. A surfactant–anthracite binary system and a water–surfactant–anthracite ternary system was constructed. Molecular dynamics simulations were performed in Materials Studio 8.0. The COMPASS force field was used to describe intermolecular interactions. NVT ensembles were used for MD simulations. Nose was used for thermostat. The time step was set to 1 fs. The temperature was 298 K. The water cell contained 2000 water molecules, and the length and width of the unit cell were consistent with the coal model. The surfactant layer contained 10 molecules. Both the water layer and the surfactant layer underwent geometric optimization and annealing algorithms to minimize energy. In all MD simulations, the Ewald algorithm was chosen for long-range electrostatic interactions, with an accuracy of 0.001 kcal/mol, and the atom-based algorithm was chosen for van der Waals interactions, with a cutoff distance of 1.25 nm. A 12 nm vacuum layer was added to the surface of each model to eliminate mirror effects. The bottom two-thirds of the anthracite model was fixed. In systems with larger atomic weights, this approach will save considerable computation time. According to Zhang et al. [45], this operation did not affect the computational results.

**Figure 2:**
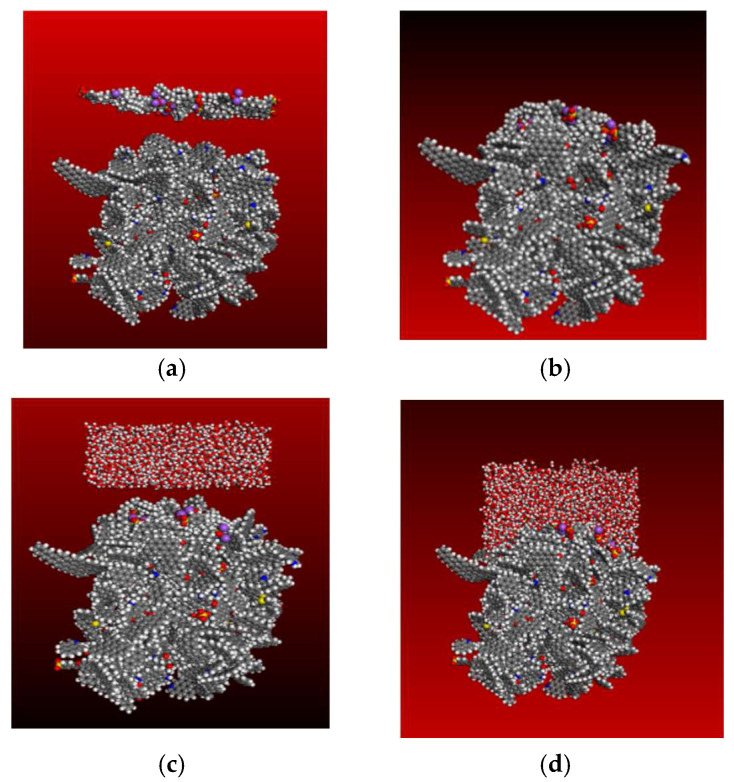
MD simulation process: (**a**) initial conformation in the first stage; (**b**) equilibrium conformation in the first stage; (**c**) initial conformation in the second stage; (**d**) equilibrium conformation in the second stage.

The MD simulation process was carried out in two stages. The first stage was the adsorption of surfactant and anthracite. In this step, the microscopic conformation and energy changes of the adsorption process can be effectively observed. The second stage was the interaction between water molecules and the modified anthracite. The duration of the first simulation was 1000ps, and the duration of the second simulation was 500ps. The adsorption of SDBS to anthracite is shown in Figure 2, and the adsorption process of STAC to anthracite is shown in the Supplementary Materials.

## 3. Results and Discussion

### 3.1. Contact Angle Analysis

**Figure 3:**
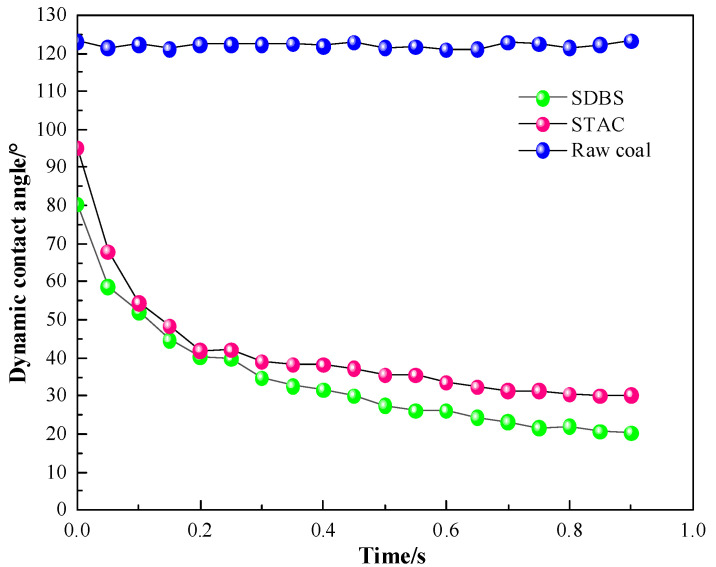
Dynamic change diagram of contact angle of surfactant and deionized water.

Contact angle is an important evaluation index, used to measure the performance of coal samples. This dynamic contact angle experiment measured the data within 1 s from the start of the surfactant contact with anthracite until the contact angle remained relatively stable. Deionized water was used as a control experiment to analyze and evaluate the adsorption mechanism of ionic surfactants on anthracite samples. The dynamic change diagram of the contact angle of surfactant and deionized water is shown in Figure 3.

**Figure 4:**
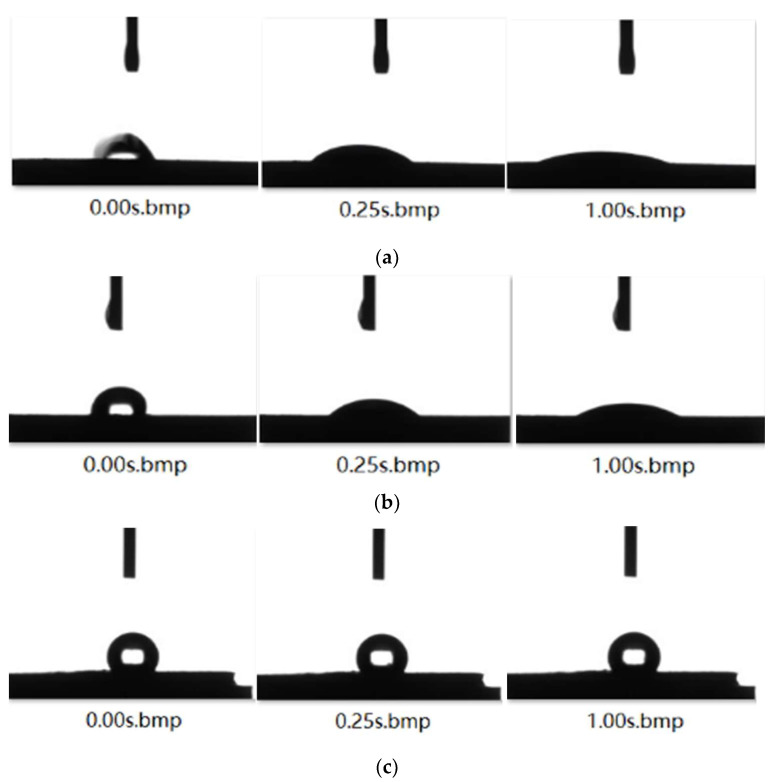
Dynamic change in contact angles of (**a**) SDBS, (**b**) STAC, (**c**) H_2_O and anthracite coal.

The contact angle between deionized water and anthracite samples has almost no change, and the contact angle value is basically stable in the range of 121~122°. As shown in Figure 4, the contact angles of the two surfactant solutions greatly changed after contact with anthracite, the contact angles rapidly changed in the first 0.25 s, and the change amplitudes of the contact angles gradually decreased in the subsequent period. In the same time interval (the first 0.25 s), the contact angle reduction efficiencies of SDBS and STAC were 50.436% and 55.824%, respectively, showing that, in the initial stage, STAC had a stronger ability to reduce the contact angle than SDBS. From 0.25 s to 1 s, the contact angle of the STAC system changed more slowly than that of the SDBS system, indicating that STAC reaches equilibrium adsorption on the surface of anthracite earlier. This is because the adsorption speed of STAC molecules on the surface of anthracite is faster than that of SDBS, and a relatively dense surfactant layer formed on the surface of anthracite within a short time, so that the STAC molecules could not continue to adsorb rapidly in the later stage.

### 3.2. XPS Analysis

**Figure 5:**
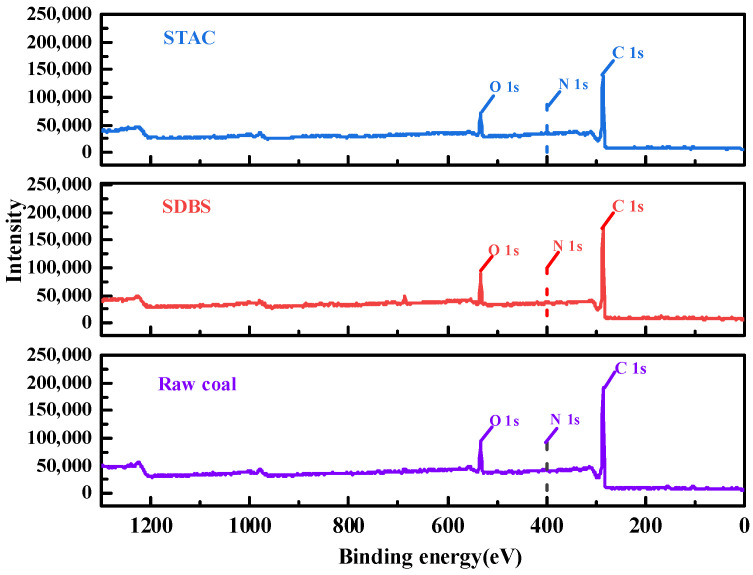
XPS spectrum of anthracite raw coal and adsorption of surfactant.

The adsorption of SDBS and STAC on the surface of anthracite was analyzed by the XPS method to explore the microscopic adsorption mechanism of ionic surfactant on the surface of anthracite. The relative atomic contents of the three main obtained elements (except H) are shown in Table 1, and their XPS spectra are shown in Figure 5.

The content of elements that changed greatly after SDBS and STAC were adsorbed on the surface of anthracite. It can be seen from Table 1 that SDBS is smaller than STAC regardless of which element of C, O, and N is considered. Firstly, the change in C element was analyzed. After adsorption, the content of the C element in STAC system increased by 2.20%, while that in SDBS system decreased by 1.47%. This is because STAC covers other element sites after a large amount of adsorption on the coal surface and exposes a large amount of the C element contained in itself, while SDBS has only a small amount of adsorption and, after adsorption, it covers the C element sites on the surface of the raw coal, exposing other elements. The change in O element shows that the decrease in STAC is greater than the increase in SDBS, because the adsorption of STAC on the surface of anthracite covers a large number of oxygen-containing functional groups on the surface of anthracite. The increase in oxygen content in SDBS system is because one SDBS molecule contains three oxygen atoms, and the oxygen content increases after being adsorbed on the surface of anthracite with very little oxygen content. The content of N element in the SDBS system is almost unchanged, which means that SDBS does not adsorb much on the coal surface and cannot cover the N atoms on the coal surface. However, STAC significantly changed, due to the fact that the STAC molecule already contains N atoms, which is caused by the large amount of adsorption on the surface of anthracite. In conclusion, STAC has a stronger adsorption capacity and denser layered structure on the surface of anthracite.

The electrostatic force between the positively charged STAC on the head base and the negatively charged anthracite is much higher than that of the negatively charged SDBS on the head base. In addition, the hydrophobic tail of STAC molecule includes 18 straight-chain alkane structures, while SDBS contains 12 straight-chain alkanes and a benzene ring structure. The benzene ring structure is more polar than the straight-chain alkane structure. The benzene ring structure will have a π-π stacking effect with a large number of aromatic structures in anthracite, and the polar interaction will reduce the non-polar interaction between SDBS and coal to a certain extent, Therefore, STAC will undergo stronger hydrophobic bonding with the non-polar part of the coal surface than SDBS [46]. Self-polymerization also occurs between the hydrophobic groups of the STAC molecule itself, forming a self-polymer with a large degree of aggregation [47,48,49]. The adsorption strength of STAC on the surface of anthracite is higher than that of SDBS.

### 3.3. FTIR Analysis

**Figure 6:**
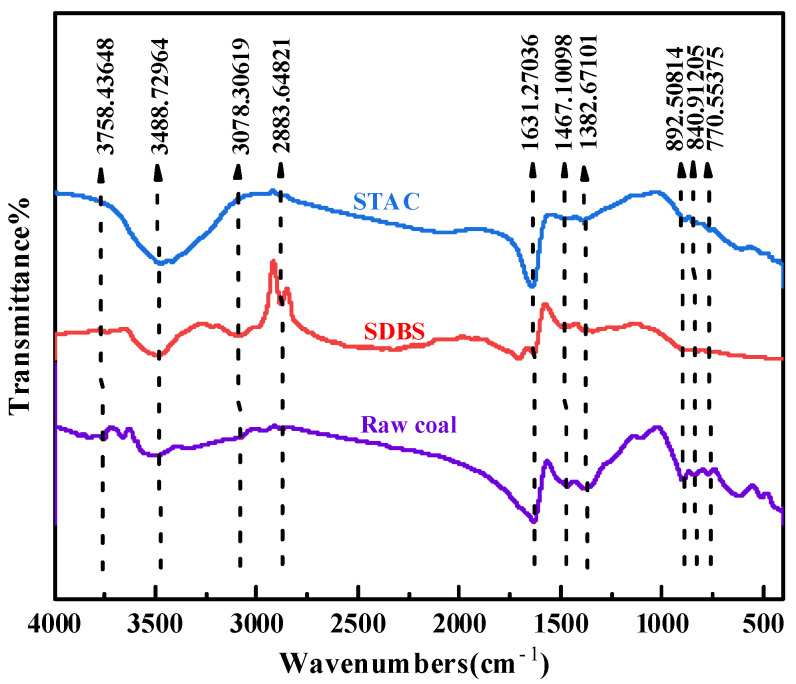
FTIR spectral map of anthracite adsorption of different surfactants.

The adsorption distribution of surfactants on the surface of anthracite was analyzed by Fourier transform infrared spectroscopy (FTIR). The results are shown in Figure 6. The infrared absorption peaks in the samples are divided into three types, among which the peaks in aromatic structural units are mainly located at 600–900 cm^−1^, the peaks in oxygen-containing functional groups are mainly located at 900–1800 cm^−1^, and the peaks in hydroxyl structural units are located at 3000–3700 cm^−1^ [50]. Side chain groups are almost absent, consistent with the structural characteristics of anthracite.

Figure 6 shows that the samples adsorbed by SDBS and STAC have a strong peak in the hydroxyl stretching vibration range, which is located at 3488 cm^−1^ [16,51,52]. The peaks in the two samples are relatively obvious, but the peak area of the SDBS adsorption sample is much smaller than that of STAC, and the hydrogen in the water molecule associates with the oxygen sites in the oxygen-containing functional groups on the coal surface, forming a high number of hydrogen bonds [23,39,53]. The adsorption capacity of STAC on the surface of anthracite is higher, the adsorption strength is higher, and the content of the formed hydrogen bonds is higher, resulting in the content of -OH bonds in the STAC system being higher than that in the SDBS system. It belongs to the range of -CH_2_ bond symmetric stretching vibrations and asymmetric stretching vibrations at 2883 cm^−1^ [16,52,53]. Within this range, the peak area of the SDBS system is much smaller than that of STAC system, which is because STAC has a larger adsorption capacity on the surface of anthracite, and the adsorption is denser. The vibrational peak in the aromatic ring is around 700–900 cm^−1^ [16]. The surface of the two samples and the raw coal after adsorbing the surfactant showed three weak and extremely small peaks, and the peak area was smaller than that of the raw coal. Three weak peaks appeared on the surface of the modified coal, and the peak areas were smaller than those of the raw coal. This phenomenon indicates that the SDBS-containing aromatic ring structure has a low amount of adsorption on the surface of anthracite. The reduction in the content of aromatic ring structures on the coal surface after the adsorption of STAC indicates that STAC is largely covered on the surface of anthracite, which also reflects its strong adsorption capacity on the coal surface [54]. In conclusion, STAC can be more effectively adsorbed on the surface of anthracite than SDBS. This result is consistent with the XPS analysis result.

### 3.4. Molecular Dynamics Simulation

#### 3.4.1. Contact Surface Area

The contact surface area (CSA) can reflect the adsorption strength between two materials. The contact surface area is positively correlated with the adsorption strength, which is defined by Formula (2).

(2) $${\mathrm{CSA}=(\mathrm{SASA}}_{\mathrm{anthracite}}\;{+\;\mathrm{SASA}}_{\mathrm{surfactant}}-{\;\mathrm{SASA}}_{\mathrm{total}})\;/\;2$$

where SASA_anthracite_, SASA_surfactant_, and SASA_total_ are the solvent-accessible surface area (SASA) of the anthracite model, the surfactant, and the anthracite–surfactant binary model, respectively. In this investigation, the applicable probe radius was 0.14 nm.

The CSA of the STAC system is 33.31 nm^2^, and the CSA of the SDBS system is 31.03 nm^2^. The CSA after adsorption of STAC is larger than that of SDBS, indicating that the adsorption of STAC and anthracite is more closely linked. This is because the STAC is positively charged and has a strong electrostatic attraction to the negatively charged coal surface.

#### 3.4.2. Mass Density

**Figure 7:**
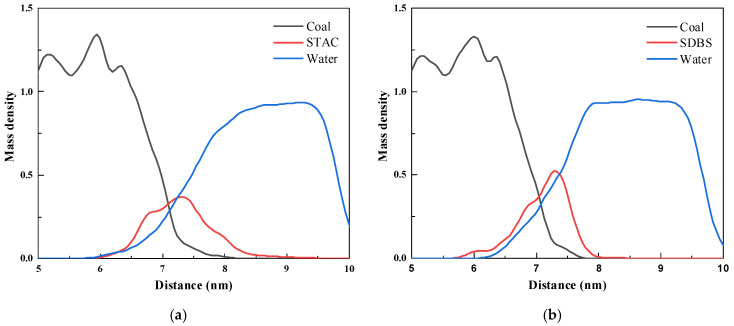
Mass density of different systems (**a**) STAC; (**b**) SDBS.

As seen from Figure 7 that STAC is distributed in the range of 6.04–8.94 nm, with a width of 2.90 nm, and a peak in 0.37, which was was obtained at 7.34 nm; SDBS is distributed in the range of 5.80–7.95 nm, with a width of 2.15 nm and a peak of 0.52 at 7.28 nm. The distribution range of STAC is wider and the peak value is smaller, indicating that its layered structure is more uniformly distributed on the coal–water interface. The starting point of the water layer in STAC is 5.96 nm, and the starting point of the water layer in SDBS is 6.23 nm. The presence of SDBS causes the water layer to retreat. This is due to the existence of the benzene ring structure in SDBS, which produces a π-π stacking effect with a high number of aromatic structures in anthracite, which makes SDBS closer to the surface of the coal, producing an “isolation” effect on the water layer to a certain extent.

#### 3.4.3. Adsorption Energy

The adsorption energy is defined by the Formulas (3)–(5).

(3) $${\mathrm{EV}=\mathrm{EV}}_{\mathrm{total}}-{\;\mathrm{EV}}_{A}-{\;\mathrm{EV}}_{B}$$

(4) $${\mathrm{EE}=\mathrm{EE}}_{\mathrm{total}}-{\;\mathrm{EE}}_{A}-{\;\mathrm{EE}}_{B}$$

(5) E=EV+EL

where EV stands for van der Waals adsorption energy, EE stands for electrostatic adsorption energy, E stands for total adsorption energy, E_total_ represents the total energy after the adsorption of the two materials is completed, E_A_ represents the energy of material A, and E_B_ represents the energy of material B.

The adsorption energy of the two systems are shown in Table 2.

Comparing the total adsorption energy between the modified coal surface and water molecules, the energy released by the STAC system is much higher than that of the SDBS system, indicating that the interaction force between the coal surface and water molecules is stronger after being adsorbed by STAC, and the binding ability to water molecules is stronger. The van der Waals adsorption energy and electrostatic adsorption energy were further analyzed, and the adsorption energy of the STAC system is almost entirely contributed by the electrostatic adsorption energy. This is due to the strong electrostatic attraction between the head base band of STAC and the negatively charged oxygen atoms in water. In the SDBS system, the electrostatic force is dominant, and the van der Waals force accounts for about 5%.

#### 3.4.4. Mean Square Displacement

The MSD of water molecules in both systems was calculated, and the diffusion coefficient D of water molecules was calculated by Formula (6):

(6) $$D=\underset{t\to \infty }{\lim\;}\left(\frac{\mathrm{MSD}}{6t}\right)=\frac{1}{6}\;K$$

The diffusion coefficient of STAC is 4.66 × 10^−9^ m^2^/s and the diffusion coefficient of SDBS is 5.31 × 10^−9^ m^2^/s. The diffusion coefficient of water molecules in the STAC system is lower than that of the SDBD system, indicating that the surface of the coal modified by STAC have a stronger binding capacity with water molecules. This is consistent with the adsorption energy analysis results presented above.

## 4. Conclusions

(1) By measuring the contact angles of the two surfactants on the surface of anthracite and comparing with deionized water, it is concluded that STAC has stronger adsorption capacity on the surface of anthracite.
(2) Through XPS experiment, the element changes in the SDBS system and STAC system were compared with those of raw coal system, and it was concluded that the adsorption capacity of STAC on the surface of anthracite was much higher than that of SDBS. Moreover, STAC forms a denser surfactant layer on the surface of anthracite through stronger electrostatic interaction, hydrophobic bonding and self-polymerization, which better covers the surface of the coal, so that the adsorption strength of STAC on the surface of anthracite is higher than that of SDBS.
(3) The functional groups and chemical bond forms on the surface of the samples after adsorption were analyzed by FTIR experiments, and it was found that the adsorption efficiency of STAC on the surface of anthracite is better than that of SDBS, and the strength after adsorption is higher than that of SDBS.
(4) Through a molecular dynamics’ simulation, CSA, MSD, adsorption energy and mass density were calculated. The study showed that STAC is more closely adsorbed on the surface of anthracite, and the distribution is more uniform at the coal–water interface. The surface of anthracite modified by STAC has a stronger binding ability to water molecules.

## Figures and Tables

**Table 1:** The relative content of the atomic number of the main elements (except H) on the surface of anthracite after adsorption.

Samples	Relative Atomic Number of Major Elements (%)		
	C	O	N
Raw Coal	85.65	13.30	1.05
SDBS	84.18	14.78	1.04
STAC	87.85	10.45	1.70

**Table 2:** Adsorption energy between water and raw or modified anthracite surface.

Model	EV/(kcal·mol^−1^)	EE/(kcal·mol^−1^)	E/(kcal·mol^−1^)
Water–STAC–anthracite	−0.14	−5329.68	−5329.82
Water–SDBS–anthracite	−88.53	−1571.70	−1660.23

## Data Availability

The raw/processed data required to reproduce these findings cannot be shared at this time as the data also form part of an ongoing study.

## Supplementary Materials

The following supporting information can be downloaded at: https://www.mdpi.com/article/10.3390/molecules27165314/s1, Figure S1: MD simulation process of STAC.

## Sample Availability

Samples of the anthracite is available from the authors.
