# Supplementary figures and images for: Adsorption Characteristics of Ionic Surfactants on Anthracite Surface: A Combined Experimental and Modeling Study
Source: Molecules. 2022 Aug 20;27(16):5314. doi: 10.3390/molecules27165314 (PMC9416174; doi:10.3390/molecules27165314)

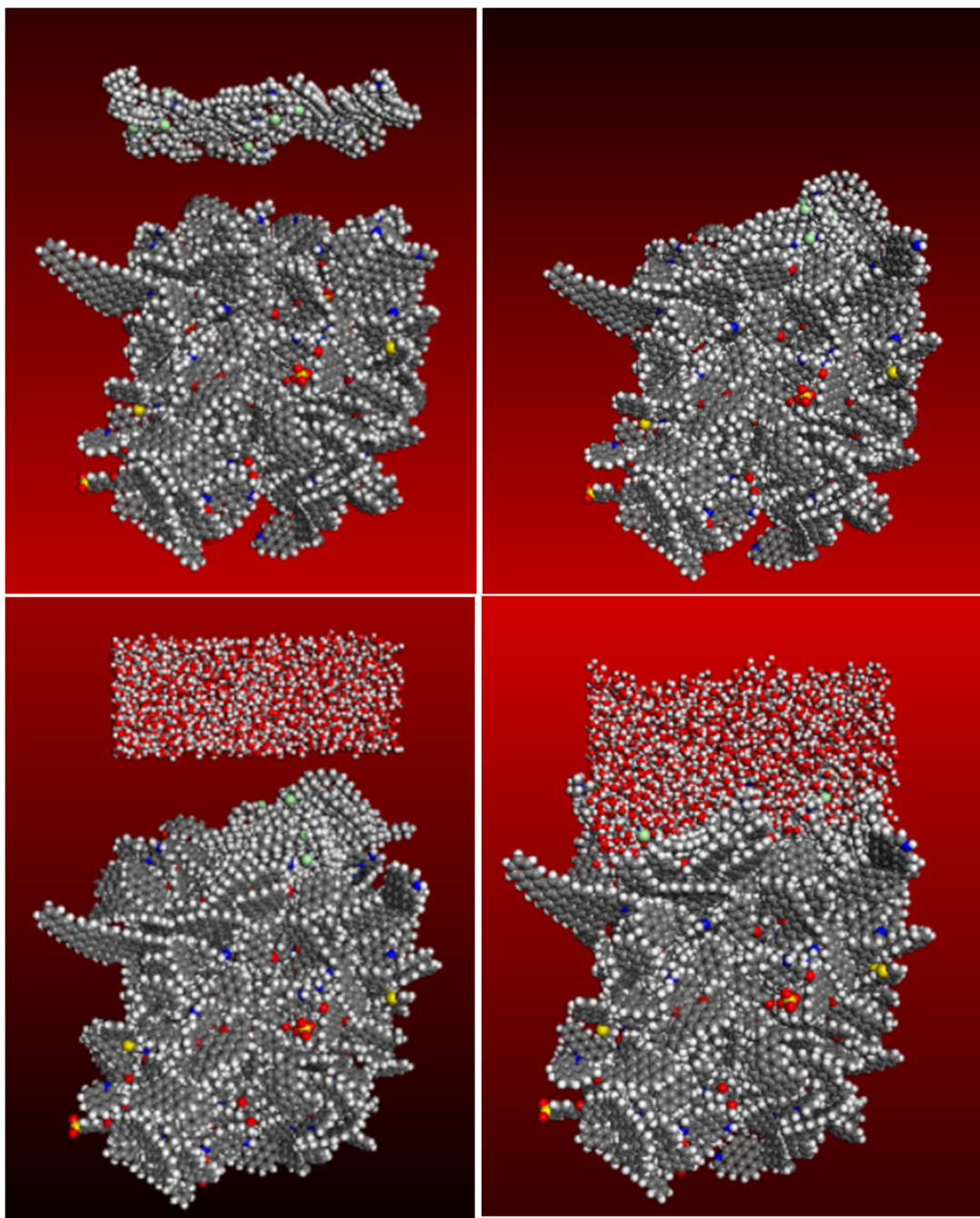

**Figure S1.** MD simulation process of STAC.

Supplement: Supplementary file 1 [file molecules-27-05314-s001.zip › molecules-1855431-supplementary.pdf]
